# The potential effects of anabolic-androgenic steroids and growth hormone as commonly used sport supplements on the kidney: a systematic review

**DOI:** 10.1186/s12882-019-1384-0

**Published:** 2019-05-31

**Authors:** Dorna Davani-Davari, Iman Karimzadeh, Hossein Khalili

**Affiliations:** 1 Department of Clinical Pharmacy, Faculty of Pharmacy, Shiraz University of Medical Sciences, Karafarin street, P O Box: 7146864685, Shiraz, Iran; 2 Department of Clinical Pharmacy, Faculty of Pharmacy, Tehran University of Medical Sciences, Tehran, Iran

**Keywords:** Sport supplements, Growth hormone, Anabolic-androgenic steroids, Kidney, Adverse effects

## Abstract

**Background:**

Anabolic-androgenic steroids and growth hormone are among the most commonly used supplements by sportsmen and sportswomen. The aim of this systematic review is to collect and report available data about renal safety of anabolic-androgenic steroids and growth hormone (GH).

**Methods:**

The search strategy was in accordance with the PRISMA guideline. Seven databases such as Scopus, Medline, Embase, and ISI Web of Knowledge were searched using keywords, such as “growth hormone”, “anabolic-androgenic steroids”, and “kidney injury”. Articles published from 1950 to December 2017 were considered. Randomized clinical trials, prospective or retrospective human studies, case series as well as case reports, and experimental (in vivo) studies were included. Twenty one clinical and experimental articles were selected (12 for anabolic-androgenic steroids and 9 for GH).

**Results:**

Anabolic-androgenic steroids can affect the kidney in different aspects. They can induce or aggravate acute kidney injury, chronic kidney disease, and glomerular toxicity. These adverse effects are mediated through pathways such as stimulating renin-angiotensin-aldosterone system, enhancing the production of endothelin, producing reactive oxygen species, over-expression of pro-fibrotic and pro-apoptotic mediators (e.g., TGF-β1), as well as inflammatory cytokines (e.g., TNF-α, IL-1b, and IL-6). Although GH may affect the kidney in different aspects, such as size, glomerular filtration rate, and tubule functions, either directly or indirectly, there is no conclusive clinical evidence about its detrimental effects on the kidney in athletes and body builders.

**Conclusion:**

Evidence regarding effects of anabolic-androgenic steroids exists; However, GH’s exact effect on the kidney at doses used by athletes and body builders has not yet been clarified. Cohort clinical studies with long-term follow-up are warranted in this regard.

## Background

For the first time in the literature in 1991, at an international Consensus Conference related to the International Olympic Committee, a statement began to become bold: “Diet significantly influences exercise performance” [1]. However, it has been reported that the ancient Egyptians, Greeks, and Romans used performance enhancing drugs for the first time in history [2].

In 2016, a total amount of $5.67 billion was spent for dietary supplements and related nutrition products in the United States (US) [3]. According to the Lieberman et al. study on 1248 US college students, supplements including multivitamins/multiminerals [42%], vitamin C [18%], protein/amino acids [17%] and calcium [13%] were used to improve muscle strength in 20% of supplement consumers. Performance enhancement (19%) and elevation of endurance level (7%) were other intensions of using supplements in this study [4]. In 2014, statistics indicated that protein supplements (41.7%), energy drinks and shots (28.6%), creatine (14.0%), amino acids (12.1%), multivitamins with caffeine (5.7%), beta-hydroxy-beta-methylbutyrate (0.2%), dehydroepiandrosterone (0.1%), and an unspecified mix of “testosterone boosters” (1.6%) were commonly used among 21,000 US college athletes [5]. Anabolic-androgenic steroids are frequently used by bodybuilders and weightlifters as dietary supplements [6].

Although supplements are commonly utilized by athletes for improving lean body mass and muscle strength, it may be plausible that they can be harmful for human health. Since kidney is a crucial site for both the metabolism and excretion of exogenous substances, it may be adversely affected by sport supplements. In this regards, for example, Daher et al reported that a series of 16 subjects with kidney complications, including acute kidney injury (AKI) had been admitted into two referral hospitals in Brazil secondary to excessive and prolonged use of veterinary intramuscular injection supplements of vitamin A (20,000,000 IU), D (35,000,000 IU) and E (6,000 IU) [7]. In a case series from 20 Iranian male body-builders, toxic hepatitis secondary to chronic ingestion of dietary supplements including creatine and testosterone were documented [8]. However, to the best of our knowledge, there is no published article or official report about possible adverse effects of dietary supplements on the kidney in Iranian athletes especially their well-known Olympic weightlifters. Even if it has been studied, that the results may be largely negative.

The aim of this review is to collect available experimental and clinical data about renal safety of anabolic-androgenic steroids and growth hormone, as two prominent hormonal sports supplements.

## Methods

**Fig. 1:**
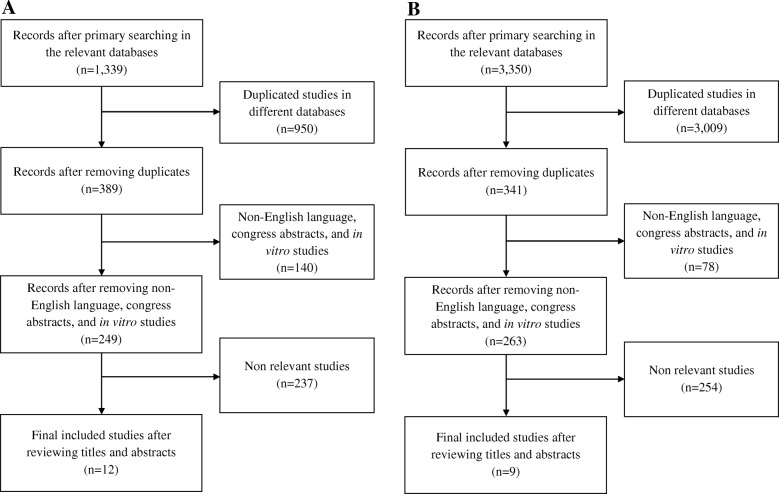
Flow diagram of study selection for anabolic-androgenic steroids (**a**) and growth hormone (**b**)

This systematic review was prepared according to the PRISMA (Preferred Reporting Items for Systematic reviews and Meta-Analyses) guideline [9]. A literature search was performed in 7 relevant databases, including Scopus, Medline, Embase, ISI Web of Knowledge, Cochrane central register of controlled trials, Cochrane database systematic reviews, and Google Scholar. To confirm consistency and accuracy of results, searches were performed by two authors independently (DDD & IK). At 3 stages, titles, abstracts, and full text of studies were evaluated. At the final stage, required data was extracted from the selected articles. The following keywords were used as search terms: “anabolic steroids”, “androgenic steroids”, “anabolic-androgenic steroids”, “androgens”, “anabolics”, “growth hormone”, “recombinant human growth hormone”, “somatropin”, “acute kidney injury”, “chronic kidney disease”, “renal dysfunction”, “renal impairment”, “renal damage”, and “renal insufficiency”. Randomized clinical trials, prospective or retrospective human studies, case series as well as case reports, and experimental (in vivo) studies were included in this review. The reference lists of published articles were also examined for identifying any additional relevant studies. Regarding publication date, articles published from 1950 to December 2017 were considered in this review. Non-English language articles, congress abstracts, newspaper articles, and in vitro studies were not eligible for inclusion. The studies included in the systematic review were reviewed by all the authors to ensure that they met the inclusion criteria. Any possible discrepancies were discussed by the authors. By taking into account the above inclusion and exclusion criteria, 21 published articles were considered in our review. These articles included experimental studies (*n* = 8), case report or case series (n = 8), pilot clinical trial (*n* = 3), placebo-controlled, cross-over clinical trial (*n* = 1), and randomized, cross-over clinical trial (n = 1). Quality of clinical studies was evaluated using the Jadad score. This score for the studies concerning growth hormone (*n* = 5) ranged from − 1 to + 2. Figure 1 depicts the flow diagram of our study selection process. 1327 and 3341 studies relevant to anabolic-androgenic steroids and growth hormone respectively, were excluded from this systematic review. This exclusion was mainly due to duplication in different databases.

## Discussion

### Anabolic-androgenic steroids

A statistics in 2007 showed that 67% of athletes in the US utilized anabolic-androgenic steroids [10]. Not only athletes, but also in the general population, especially youngsters used these agents to enhance their muscular appearance [11]. An investigation in Brazil showed that more than 25 compounds, including anabolic substances (such as Durasteron [testosterone], Stradon P [testosterone+ stradiol], Deca-durabolin [nandrolone], Uniciclo [algestone +stradiol] and Premarim [estrogens]) have been used by young bodybuilders to improve and accelerate their muscle mass gains [12]. Anabolic-androgenic steroids abuse has become particularly prevalent in regions, such as Scandinavia, the US, Brazil, and British Commonwealth countries [13]. Interestingly, a survey in 2006 revealed that only 38% of interviewed athletes were aware of side effects related to anabolic steroids [14]. Commercial magazines relevant to body builders may downplay side effects of anabolic-androgenic steroids, including cholestatic liver injury, testicular atrophy, sexual dysfunction, and, age-related cardiovascular disease.

Historically, anabolic-androgenic steroids were utilized for the treatment of anemia instead of erythropoietin in individuals with chronic kidney disease (CKD). Their doses for anemia treatment was generally lower than their doses in doping [15]. However, the use of androgens for the treatment of anemia in CKD patients has been stopped because of inconsistent erythropoietic response, many adverse effects, and the availability of recombinant erythropoietin as a more effective and safer agent [16]. Currently, anabolic-androgenic steroids are used by both elite professionals and amateur athletes to improve body image (through an increase in muscle and/or decrease in fat mass) and also exercise performance [17].

In addition to well-defined and –known adverse effects of androgens, including acne, virilization, priapism, testicular atrophy, gynecomastia, liver dysfunction, injection-site pain, peliosis, hepatitis, and hepatocellular carcinoma [18], they can also cause kidney dysfunction which is not well described. Mostly, kidney complications occur after long-term administration of anabolic-androgenic steroids. They range from a single increased serum creatinine to AKI as a complication of rhabdomyolysis or liver damage (bile acid nephropathy or cholemicnephrosis), renal histological changes, such as focal segmental glomerulosclerosis (FSGS), tubular atrophy, and interstitial fibrosis [19]. The detailed description of acute and chronic adverse effects of anabolic-androgenic steroids on renal function has been provided in the following sections.

#### Acute kidney injury (AKI)

In 2009, Daher et al. reported the case of a 21-year-old male athlete, admitted to the emergency department with complaints of nausea, progressive abdominal pain, dizziness, vomiting, headache, weakness, fever, and profuse sweating in the last month. Nausea and vomiting were in association with oliguria and arterial hypertension (160/120 mmHg). The results of laboratory tests upon admission indicated an increase in the serum levels of calcium (13.2 mEq/L), creatinine (3.9 mg/dL), and urea (79 mg/dL). The urinary analysis indicated leukocyturia (+++), hematuria (+), and proteinuria (traces). The amount of protein in the 24-h urine was 259 mg. The kidney size and renal arteries were normal. The renal biopsy showed moderate interstitial inflammatory infiltrates with eosinophils, calcium deposits, tubular necrosis, and interstitial edema. History-taking revealed that the patient was receiving anabolic steroids and veterinary supplements containing vitamin A, vitamin D, and vitamin E, respectively (20,000,000, 35,000,000, and 6000 IU, respectively). All causative agents were discontinued, and hypertension and hypercalcemia were controlled via pharmacotherapy. The patient was discharged with almost normal renal function after 20 days. In this study, the case of a 30-year-old man, experiencing nausea, vomiting, diarrhea, and fever in the past month before admission, was also reported. He was referred to the emergency department with the main complaint of persistent vomiting. The physical examination revealed the patient’s good general condition, except for abdominal pain upon palpation. According to the laboratory tests upon admission, the serum levels of urea (52 mg/dl), creatinine (1.9 mg/dl), and calcium (11 mEq/l) increased. The urinalysis showed blood (+) and protein (++). Also, the urinary level of calcium was 390 mg/24 h. The kidney size was normal. The patient confirmed the use of anabolic steroids and veterinary supplements containing vitamin A, vitamin D, and vitamin E (20,000,000, 35,000,000, and 6000 IU, respectively) in the past two years before admission. In addition, the patient was receiving 12 mg of dexamethasone every two weeks. Despite intravenous hydration and administration of furosemide and prednisolone (1 mg/kg per day), renal function did not improve. The renal biopsy revealed mild interstitial lymphmononuclear inflammatory infiltrates with eosinophils, without any remarkable tubular abnormalities. After discontinuing the use of these agents, the patient’s renal function gradually recovered after one month of hospitalization [20]. The authors also briefly described 14 other cases of AKI and relevant complications (e.g., cholestasis and rhabdomyolysis) associated with anabolic steroids (e.g., metandienone, stanozolol) and vitamin supplements (e.g., vitamins D and A) in males and females aged between 21 and 63 years published in the literature between 1988 and 2009. They demonstrated that apart from potential adverse effects of anabolic steroids on the kidney, interstitial nephritis, hypercalcemia, and nephrocalcinosis secondary to vitamin D intoxication were also capable of inducing renal dysfunction in these cases. Rhabdomyolysis has been reported in the setting of AKI due to anabolic-androgenic steroids and it can independently induce or aggravate AKI caused by these agents [20]. Nephrocalcinosis secondary to exogenous vitamin D intoxication in a bodybuilder athlete has been described in another case report [21].

Almukhtar et al. in 2015 also reported 4 bodybuilders referred to the nephrology department of a university hospital with the chief complaint of weakness and lethargy. All patients had taken more than 400 mg/week testosterone proprionate and/or nandrolone decanoate intramuscularly. They also had consumed supplementary proteins (containing 78–104 g of whey powder added to regular dietary protein including 2–3 L of milk to reach 278–354 g daily) and creatine (15 g per day). Their serum creatinine and estimated glomerular filtration rate (eGFR) were 229.84–335.92 μmol/L and 0.37–0.57 mL/s, respectively. Renal biopsy revealed acute tubular necrosis. By discontinuing all the above agents, serum creatinine became normal within four weeks. The authors attributed AKI in the bodybuilders to the combination effects of excess creatine and protein with steroid injections along with hypervitaminosis D and phosphate nephropathy [22].

Bile acid nephropathy, also known as cholemic nephrosis, can be typically associated with AKI. At least 3 case reports published between 2014 and 2016 described bile acid nephropathy secondary to cholestatic jaundice caused by anabolic steroids in bodybuilders with no underlying liver or kidney diseases. The abused anabolic steroids in the above case reports were oral or injectable stanazolol, injectable nandrolone, injectable testosterone, and oral methandrostenolone consumed for 5 to 6 weeks or oxandrolone, boldenone undecyclenate, stanazolol, and trenabol for an unidentified duration. Hyperbilirubinemia, increased serum creatinine, oliguria and tubular bile acid casts in urine specimen were observed in the cases. All AKI episodes were resolved by either only discontinuing the offending medication or along with supportive care and renal replacement therapy [19, 23, 24].

Regarding the effects of endogenous sex hormones on the urinary markers of nephrotoxicity, an experimental study in rats demonstrated that there was a significant association between testosterone and urinary excretion of leucine aminopeptidase, alkaline phosphatase, γ-glutamyl transpeptidase, cystatin C and β2-microglobulin, as biomarkers of kidney’s proximal tubule [25]. The authors stated that these data should be considered in the accurate interpretation of studies about markers of nephrotoxicity in animals.

#### Chronic kidney diseases (CKD)

Endocrine dysfunctions, such as testosterone deficiency and hypogonadism may occur in male patients with CKD [26]. These dysfunctions are associated with a higher risk of morbidity and mortality, possibly due to anemia, mineral as well as bone disorders (osteoporosis & osteodystrophy), and cardiovascular diseases in CKD individuals [27]. Conversely, exogenous testosterone administration can also cause renal dysfunction, renal injury progression, and proteinuria [28]. Anabolic-androgenic steroids can cause or exacerbate CKD and also kidney fibrosis or sclerosis with different mechanisms:

##### Renal blood pressure regulation

Since androgen receptors are located in different parts of the kidney and on the other hand, various enzymes involved in testosterone synthesis pathway are produced in the kidney tissue, it is conceivable that testosterone is able to regulate renal artery blood pressure (BP) [27].

Testosterone can increase renal artery BP, probably via potentiating the renin–angiotensin-aldosterone system (RAAS) along with the up-regulation of endothelin. RAAS can increase BP and water retention through promoting tubular sodium and water re-absorption [29, 30]. An experimental investigation in male rats revealed that androgens potentiate Ang II-induced renal vascular responses, partly via up-regulation of the Rho kinase signaling pathway [31]. Rho kinase signaling pathway can increase the resistance of peripheral vessels, leading to BP elevation [32]. This signaling pathway is involved in the pathogenesis of CKD [31]. Interestingly, a study in hypertensive female rats under high-sodium diet revealed that exogenous testosterone is involved in development of hypertension [33]. Furthermore, a study on orchidectomized adult male Sprague-Dawley rats demonstrated that a subcutaneous injection of testosterone for 7 days with a dose of 125 mg/kg/day or 250 mg/kg/day can increase water re-absorption. A resulting effect can be rise in BP by the expression of aquaporin types 1&7 in the proximal convoluted tubule and 2, 4&6 in the collecting ducts [34]. Paradoxically, low levels of endogenous testosterone can also lead to high BP [35]. In this regards, men with hypertension had lower levels of testosterone compared with normotensive ones of the same age [36]. In addition, restoration of testosterone levels to normal range in hypogonadal men decreased BP [37]. The exact explanation for these paradoxical effects of androgens on BP was unknown.

##### Endothelin

Testosterone can enhance the production of endothelin directly or indirectly (via RAAS) [30, 38]. Endothelin can result in vasoconstriction (both afferent and efferent arterioles) and promotion of mitogenic activity through its type A receptor. This receptor is exclusively expressed by vascular smooth muscle cells in the kidney. The role of endothelin in pathophysiological conditions, such as diabetic nephropathy, and immune nephritis has been also implicated [39]. Therefore, testosterone may be involved in both kidney fibrosis and ischemia-reperfusion injury via both local and systemic effects of endothelin [27].

##### Oxidative stress

Anabolic-androgenic steroids may play a role in the development of CKD via producing reactive oxygen species and promoting oxidative stress [40]. In this regards, orchidectomy has been demonstrated to attenuate oxidative stress-mediated kidney fibrosis and proteinuria after ureteral obstruction in male rats [41]. Testosterone can cause oxidative stress directly, as well as indirectly via activation of the RAAS and endothelin (through up-regulating NADPH oxidase) [27].

##### Apoptosis & Inflammatory cytokines

Apoptosis, another contributory factor in kidney fibrosis development, can be induced by various inflammatory cytokines. Androgens play a crucial role in apoptosis. For example, androgens can induce apoptosis of renal tubular cells through triggering a caspase-dependent apoptotic pathway [42]. In addition, testosterone may be involved in the production of pro-inflammatory cytokines such as, tumor necrosis factor a (TNF-α), interleukin-1b (IL-1b), and interleukin-6 (IL-6). The production of such cytokines can lead to renal inflammation and CKD progression [43–45]. Reciprocally, inflammatory cytokines, such as TNF-α and IL-6 can enhance the activity of androgen receptors [46, 47]. Accordingly, Metcalfe et al. demonstrated that TNF-α production, pro-apoptotic, as well as pro-fibrotic signaling and consequently, level of tubule-interstitial fibrosis and kidney dysfunction were increased in normal male rats. This increase can lead to endogenous production of testosterone and oophorectomized female rats treated by exogenous testosterone [28]. Moreover, exogenous administration of testosterone has shown to induce podocyte apoptosis and glomerulosclerosis in female estrogen receptor-knockout mice [48].

#### Progression of CKD

A number of investigations have shown a relation between the male gender and multiple kidney disorders, such as IgA nephropathy, polycystic kidney disease, and membranous nephropathy. Therefore, androgens can be involved in the progression of CKD [49–51]. Accordingly, in diabetic nephropathy, male gender is a risk factor for proteinuria progression [52]. In contrast, some evidence suggested that an imbalance in sex hormones’ ratio (rather than androgen excess alone) may cause or aggravate kidney dysfunction. For example, Maric et al. demonstrated that lower levels of endogenous testosterone and higher levels of blood estradiol were associated with the development of diabetic nephropathy in men [53]. Administration of exogenous testosterone and aromatase inhibitors can restore dihydrotestosterone and estradiol levels to their physiological range. Consequently, they can even act as renal protective agents in the progression of diabetic nephropathy via reducing inflammatory process and fibrosis [54, 55].

Xu et al. demonstrated a dose-dependent relation between administration of exogenous dihydrotestosterone and albuminuria, glomerulosclerosis, and tubule-interstitial fibrosis progression in castrated male diabetic rats. Administration of 0.75 mg/day dihydrotestosterone (low dose) had nephroprotective effects; whereas, administration of 2.0 mg/day dihydrotestosterone (high dose) accelerated renal injury process. Additionally, estradiol could affect the dose-dependent action of dihydrotestosterone on the kidneys [56].

#### Glomerular toxicity

Herlitz et al. in 2010 described variable degrees of renal insufficiency, proteinuria, and nephrotic syndrome in 10 bodybuilders with the mean age of 37 years abusing anabolic steroids. At least one anabolic-androgenic steroid, usually combined with dietary supplements (e.g., monohydrate, creatine, and a high-protein diet). They manifested along with proteinuria and renal insufficiency (mean creatinine level, 3.0 mg/dl). Nephrotic syndrome was detected in three out of 10 (30%) patients. According to the renal biopsy, FSGS and ≥ 40% tubular atrophy and interstitial fibrosis were found in nine and three patients, respectively. Among all seven patients with long-term follow-ups, discontinuation of anabolic steroids, along with the use of RAAS blockers and/or corticosteroids, has led to the improvement or stabilization of serum creatinine, weight loss, and proteinuria reduction. The authors hypothesized that secondary FSGS in anabolic-androgenic steroid abusers may be related to different pathways: 1) an increase in lean body mass which may result in glomerular hyperfiltration; 2) overexpression of a potent profibrotic and proapoptotic cytokine (TGF-β1); 3) induction of oxidative stress; and 4) upregulation of RAAS components. The last three mechanisms can be attributed to the potential toxic effects of anabolic-androgenic steroids on glomeruli. Besides these mechanisms, other factors including high-protein diet (by increasing the renal blood flow and GFR) and elevated blood pressure (via hypertensive arterionephrosclerosis) may have additive/synergistic adverse effects on glomeruli [57].

One year later, Harrington et al. reported another case of secondary FSGS caused by anabolic steroid abuse in a 38-year-old man. History taking revealed regular use of anabolic steroids, both orally and intramuscularly since the age of 18. Para clinical evaluation demonstrated high serum creatinine (1797 μmol/l), increased serum urea concentration (55.2 mmol/l), low hemoglobin level (6.0 g/l), intrinsic renal parenchymal damage, and FSGS. The patient required renal replacement therapy due to his end-stage renal disease (ESRD). Hemodialysis and after that, continuous ambulatory peritoneal dialysis were initiated for him [58]. It is noteworthy that these two reports are just case descriptions and obviously, not epidemiological studies. A summary of published experimental and clinical studies regarding renal safety of anabolic-androgenic steroids is shown in Table 1 in the order of study type (first experimental and after that clinical) and publication year.

**Table 1:** Summary of experimental and clinical studies about the renal safety of anabolic-androgenic steroids (*n* = 12)

Dose & Duration	Subjects	Type of study	Main results	Reference
50 mg/day dihydrotestosterone intraperitoneally for 10 days	Rats	Experimental	- Increase in blood pressure, and proximal tubule volume reabsorption - Decrease in serum angiotensin II level - No change in glomerular filtration rate	Quan et al. 2004 [29]
500 μg/kg/day testosterone propionate intramuscularly for 2 weeks	Castrated male and oophorectomized female rats with obstructive renal injury	Experimental	Increase in TNF-α production and pro-apoptotic and pro-fibrotic signaling leading to increased apoptotic cell death, tubulointerstitial fibrosis, and renal dysfunction	Metcalfeet al 2008 [28]
0.75 or 2.0 mg/day dihydrotestosterone as subcutaneous implants for 14 weeks	Castrated diabetic male rats	Experimental	- Low doses attenuated castration-associated increases in urine albumin excretion, glomerulosclerosis, and tubulointerstitial fibrosis - High doses exacerbated castration-associated increases in urine albumin excretion, glomerulosclerosis, and tubulointerstitial fibrosis	Xu et al. 2009 [56]
Testosterone implants (20 mg/capsule) changed every 2 weeks	Hypertensive rats on a high sodium diet	Experimental	- Increase in blood pressure & renal sodium reabsorption - Increase in glomerulosclerosis	Liu&Ely 2011 [33]
Single dose of testosterone (12.5 mg/pellet) orally	Female estrogen receptor knockout mice	Experimental	Inducing podocyte apoptosis by androgen receptor activation, independent of the TGF-β1 signaling pathway	Doublier et al. 2011 [48]
Combination of 0.75 mg/day dihydrotestosterone as subcutaneous implants and 0.15 mg/kg/day anastrozole orally for 12 weeks	Diabetic male rats	Experimental	- Attenuating albuminuria, glomerulosclerosis, and tubulointerstitial fibrosis - Decrease in the density of renal cortical CD68-positive cells - Decrease in the expression of transforming growth factor-β, collagen type IV, TNF-α, and IL-6	Manigrasso et al. 2012 [55]
Case 1: Not defined	Case 1: 21-year-old male athlete	Case report and case series	- Arterial hypertension, oliguria, leukocyturia, hematuria and proteinuria, increase in serum urea and creatinine - Moderate interstitial inflammatory infiltrate with eosinophils, interstitial edema, calcium deposits, and mild acute tubular necrosis	Daher et al. 2009 [20]
Case 2: Not defined	Case 2: 30-year-old male bricklayer		- Increase in serum urea and creatinine, hematuria and proteinuria - Mild interstitial lymphmononuclear inflammatory infiltrate with eosinophils without remarkable tubular abnormalities	
Case series: Not defined	Case series: Males & Females aged between 21 and 63 years		- Interstitial nephritis and hypercalcemia secondary to vitamin D intoxication caused acute kidney injury	
At least one anabolic steroid (e.g., testosterone 500 mg twice weekly) intramuscularly for several months	10 body builders aged between 28 and 49 years	Case series	- Proteinuria, renal insufficiency, and nephrotic syndrome - Focal segmental glomerulosclerosis, tubular atrophy, and interstitial fibrosis	Herlitz et al. 2010 [57]
Not defined	38-year-old man	Case report	- High serum creatinine, high serum urea, low hemoglobin level - Intrinsic renal parenchymal and focal segmental glomerulosclerosis	Harrington et al. 2011 [58]
- Nandrolone intramuscular injection 400 mg twice per week for 6 weeks - Testosterone intramuscular injection 400 mg once per week for 6 weeks	41-year-old male bodybuilder	Case report	Acute kidney injury with the pathology of diffuse acute tubular injury due to bile acid nephropathy with the pathology of tubular bile acid casts	Luciano et al. 2014 [19]
Case 1: Stanozolol intramuscular injection 10 mg three times per week for 5 weeks Case 2: Stanozolol intramuscular injection 1 mg three times per week for 6 weeks	Case 1: 30-year-old male amateur bodybuilder Case 2: 43-year-old male amateur bodybuilder	Case report	Bile cast nephropathy due to cholestatic jaundice characterized by acute tubular epithelial cell damage along with increased serum creatinine and oliguria	Tabatabaee et al. 2015 [23]
Oxandrolone, boldenone undecyclenate, stanozolol, and trenabol (with not-defined daily dose and duration of treatment)	28-year-old male bodybuilder	Case report	Acute kidney injury in the setting of severe cholestatic jaundice with the pathology of bile inclusions within tubular cells and interstitial edema	Alkhunaizi et al. 2016 [24]

In brief, regular, long-term use of anabolic-androgenic steroids can induce various renal disorders directly or indirectly through different mechanisms [19]. Some mild renal abnormalities, such as increase in serum creatinine, blood urine nitrogen, or uric acid, without sclerotic/fibrotic morphological alteration or decrease in cystatin C clearance, can be recovered after discontinuing anabolic-androgenic steroids [59]. However, their consumption by some individuals may be associated with poor kidney prognosis, resulting in ESRD. As a result, well-designed clinical studies are warranted to examine the exact pathological effects and roles of different doses of endogenous or exogenous androgens on the progression of kidney dysfunction in patients with CKD.

### Growth hormone

Growth hormone (GH) treatment has been initiated since 1960s in children with impaired growth [60]. At first, this hormone was extracted from donor pituitaries. Almost in 1980s, recombinant forms of this hormone was manufactured, and its utilization was extended [61]. This agent has been currently approved by the FDA for use in GH failure or short stature, due to Turner, Prader-Willi, or Noonan syndromes, as well as, idiopathic short stature, HIV-associated cachexia, and short bowel syndrome in adults [62].

#### Growth hormone sources in the body, its biological effects, and general safety

GH is a polypeptide hormone [63]. GH genes are expressed in pituitary somatotropic cells, placenta, and to a lesser extent in lymphocytes [64]. GH expression in lymphocytes is merely adequate for local paracrine/autocrine regulations, and all physiological actions of this hormone are mediated by pituitary and placental GH [65]. The most important sites of GH metabolic clearance are the kidneys and liver [65].

GH plays a crucial role in some biological activities, including nitrogen retention, amino acid transportation into muscle, promotion of somatic growth, growth plate elongation, generation of insulin-like growth factor I (IGF-I) and insulin-like growth factor binding protein 3 (IGFBP), lipolysis, sodium or phosphorus retention, producing insulin antagonistic effects, cell hyperplasia, and lactogenesis [65]. GH can also convert T4 to T3 and active cortisone to its inactive form [66].

Due to the beneficial effects of GH on lean body mass, and performance, as well as not being detected within the body, GH abuse is very common among athletes [67, 68]. Although a systematic review in 2008 claimed that GH can elevate lean body mass [69], at least one randomized, placebo-controlled, blinded study demonstrated that this increase in lean body mass is primarily the result of the extracellular water volume expansion [70]. However, some evidence suggested that an increase in GH level may enhance physical performance, increase tolerance for hard training, and shorten recovery time after exercise [71].

Regarding safety, GH can cause a number of adverse reactions, such as muscle pain, joint stiffness and pain, paresthesia, carpal tunnel syndrome, and headache. These adverse effects may be caused through fluid retention and are generally preventable by decreasing the dose [66]. Since GH can affect calcium absorption in the intestine and increase its excretion, calcium balance may be disturbed [72]. Although some evidence have shown that GH treatment can elevate plasma insulin concentration leading to increased risk of diabetes type II [73], no evidence of high fasting glucose level and diabetes type II was observed 6 years after discontinuing GH treatment in children born small for gestational age [74]. According to these data, long-term administration of GH does not increase the risk of diabetes type 2 and metabolic syndrome [74]. Otitis media, scoliosis, slipped femoral capital epiphyses, increased risk of malignancies, and sudden death are other rare and also even unproven complications of GH treatment [75].

#### Growth hormone safety on renal functions

The functions of GH are induced directly or indirectly via synthesis of IGF-I. Since GH receptors, IGF-I, IGF-I receptors, and IGF binding proteins are expressed in the kidney tissue, GH and IGF-I can affect different aspects of this organ, such as its morphology and size, GFR, and minerals’ hemostasis [76].

GH can change the level of serum creatinine by its anabolic effects on muscles [77–79]. Although GH administration can increase GFR by about 10–15% [80], GH at the dose of 50 ng/kg/min for 2 h did not affect the GFR in healthy men [81]. Similarly, a double-blind, placebo-controlled study implicated that GH administration at the dose of 0.125 IU/kg per week subcutaneously for the first 4 weeks and 0.25 IU/kg per week for a subsequent 5 months did not increase GFR [82]. On the other hand, various studies have demonstrated elevation of GFR and renal plasma flow in patients with acromegaly [83–85].

Studies have demonstrated that GH administration in female rats [86] and dogs [87], as well as non-viral GH transmission in mice [88] resulted in the enlargement of kidneys. In line with these findings, renal parenchyma was modified in transgenic mice models by over-expressing genes coding for GH and IGF-I [89]. Wanke et al. observed that not only the mean glomerular volume, but also the number of endothelial and mesangial cells per glomerule increased in the GH transgenic mouse model of progressive renal disease compared to the control group [90]. Over-expression of IGF-I in transgenic mice caused the expansion of extracellular matrix and glomerulosclerosis [91]. In contrast to animal studies, glomerulosclerosis and renal failure are rare among patients with acromegaly [83, 92].

GH hypersecretion can increase kidney size by about 6–54% [76]. Although 7 days of GH treatment [93] or 3 days of IGF-I injection [78] did not affect human kidney size, GH administration for 6 months in individuals with GH deficiency led to an increase in its size [82, 94]. In accordance to the mentioned statement, a case report described that kidney shrinkage by about 10–20 and 20% occurred 1 and 5 months after hypophysectomy, respectively [95]. Noting that some studies have demonstrated that the kidney weight/body weight ratio is constant and this increase in the size of kidney is associated with body weight gain [86, 96].

Regarding glomerulopathy, GH hypersecretion [77] or subcutaneous injections of GH (with the dose of 2 IU in the morning and 4 IU in the evening for one week) [93] and rhIGF-I (with the dose of 60 μg/kg, at 800, 1400 and 2000 h) [78] did not significantly alter albuminuria (as an index for glomerular permeability) and β_2_-microglobin (as an index for proximal tubular involvement). However, microalbuminuria was significantly increased (but not in a pathological pattern) in acromegalic patients [79, 85], especially in those with hypertension or diabetes mellitus [97].

Extracellular volume overload, hypertension, electrolyte disorders (such as hyperphosphatemia, hypophosphaturia, hypercalciuria), and urine acidification with reduced kaliuria are other consequences of GH hypersecretion [76]. Inversely, GH treatment caused extracellular volume reduction in patients with GH deficiency [95, 98]. In addition to extracellular volume overload, GH can increase sodium and water reabsorption from renal tubules [85, 99]. It can also activate RAAS [99, 100] which may lead to hypertension.

Considering the fact that growth retardation is a common complication of CKD in children, GH has been used to treat short stature in this population, including children under conservative treatment or hemodialysis and the ones who are kidney transplant recipients [101]. A meta-analysis of 16 relevant studies (including 809 children) published from 1980 to 2011 demonstrated that apart from clinical efficacy, GH therapy did not alter kidney function (e.g., GFR) nor did it increase episodes of acute rejection, compared to placebo in children with CKD (pre-dialysis, dialysis) or transplanted kidney, respectively [102].

Overall, although GH may adversely affect different aspects of kidney such as size, GFR, and tubule functions either directly or indirectly, it has not been clarified yet whether GH at doses used by athletes and body builders can truly cause kidney dysfunction. In addition, there is no definite and conclusive clinical evidence about the detrimental effects of GH on the kidney in these populations. Details of published experimental and clinical studies about the renal safety of GH in the order of study type (first experimental and after that clinical) and publication year are summarized in the Table 2.

**Table 2:** Summary of experimental and clinical studies about the renal safety of growth hormone (*n* = 9)

Dose & Duration	Subjects	Type of study	Main results	Reference
2.5, 5, 10, and 20 lU/kg/day subcutaneously for 4–60 days	Female rats	Experimental	- Dose-dependent increase in renal weight - No change in kidney dry weight/body weight ratio - Increase in renal glomerular and tubular cell proliferation and renal DNA/protein ratio	Mehls et al. 1993 [86]
0.025, 0.1, and 1 IU/kg/day subcutaneously for 14 weeks	Male and female dogs	Experimental	- Increase in body weight gain and kidney weights - Glomerular deposits, mesangial thickening, and very slight cellular infiltration in glomeruli - Increase in the renal glomerular area - Glomerular basal lamina thickening - Increase in mesangial matrix	Molon-Noblot et al. 2000 [87]
5–10 mg/day for 3–9 days	2 patients with hypopituitarism, 1 with cirrhosis of the liver and 2 with chronic nephritis and uremia	Case report	- Decrease in plasma urea level and urea excretion - Prompt increase in creatinine clearance and phosphorus reabsorption	Gershberg 1960 [80]
More than 400 mg/week testosterone proprionate and/or nandrolone deconate intramuscularly	4 body builders aged between 20 and 26 years	Case report	- Increase in serum creatinine and decreased in eGFR - Development of acute tubular necrosis	Almukhtar et al. 2015 [22]
50 ng/kg/min as an infusion for 2 h	Healthy men	Pilot clinical trial	- Decrease in renal plasma flow - No change in GFR	Parving et al. 1978 [81]
2 IU in the morning and 4 IU in the evening subcutaneously for 1 week	Healthy men	Pilot clinical trial	- Increase in GFR and renal plasma flow - No significant change in kidney size and urinary excretion rates of albumin and β2-microglobulin	Christiansen et al. 1981 [93]
0.125 IU/kg per week subcutaneously for the first 4 weeks and 0.25 IU/kg per week for the subsequent 5 months	Growth hormone deficient adults	Double-blind, placebo-controlled, cross-over clinical trial	- No change in GFR and renal plasma flow - No effect on kidney size	Riedl et al. 1995 [82]
0·02 IU/kg/day (or 7 μg/kg/day) subcutaneously for 10 months	Adults with childhood onset GH deficiency	Pilot clinical trial	- Increase in left ventricular-mass index and kidney length - No abnormalities or change in the urine analysis	Link et al. 2001 [94]
6 IU/m^2^ per day subcutaneously for 6 days	Healthy volunteer males	Randomized, cross-over clinical trial	- Increase in the plasma renin - Increase in distal tubule sodium and water reabsorption - Decrease in mean 24-h urinary output and mean 24-h urinary sodium excretion	Hansen et al. 2001 [99]

## Conclusion

More than one-third (38.1%) of included studies about possible effects of anabolic-androgenic steroids and GH on the kidney were animal investigations. Experimental findings suggest that GH may adversely affect different aspects of kidney such as size, GFR, and tubule functions, either directly or indirectly. However, clinical data about the potential adverse effects of GH on the kidney of healthy athletes and bodybuilders is limited. Furthermore, none of the current clinical trials regarding GH were graded as high quality. In contrast to GH, anabolic-androgenic steroids, such as testosterone, dihydrotestosterone, and nandrolone have caused more prominent renal disorders ranged from a mild, reversible rise in serum creatinine and blood urine nitrogen to irreversible CKD and FSGS leading to renal replacement therapy through a number of mechanisms. They include potentiating RAAS, enhancing the production of endothelin, producing reactive oxygen species, promoting oxidative stress, inducing apoptosis and inflammatory cytokines (such as TNF-α, IL-1b, and IL-6), and over-expression of pro-fibrotic and pro-apoptotic mediators, such as TGF-β1. However, it should be noted that kidney involvement in athletes receiving anabolic-androgenic steroids can be at least partially attributed to other independent factors and mechanisms such as high-protein diet (via increase in renal blood flow and GFR), elevated blood pressure (via hypertensive arterionephrosclerosis), bile acid nephropathy (secondary to cholestatic jaundice), rhabdomyolysis, and nephrocalcinosis (secondary to exogenous vitamin D intoxication). In addition, at least one large cohort study conducted by the National Football League on 1063 retired professional football players in the US who may have taken supplements such as anabolic-androgenic steroids and GH, demonstrated that the rate of renal problems in these individuals were comparable with the general population [103]. Therefore, the above findings about anabolic-androgenic steroid adverse effects on the kidney and their relevant mechanisms reported from experimental studies along with case report as well as case series should be reproduced in at least cohort clinical studies with long-term follow-up before any definite interpretation, recommendation, and practice in this regards. The Russian athletes from the Former Soviet Union and also Iranian ones, especially the weightlifters can be suitable populations for these studies. Unfortunately, there is no published data from these populations to the best of knowledge. Finally, the defined daily dose and duration of GH and anabolic-androgenic steroids that can be used by athletes and bodybuilders with minimal concerns regarding their renal safety are other questions that should be taken into account in future clinical investigations.

## Abbreviations

AKI: Acute kidney injury
CKD: Chronic kidney disease
ESRD: End stage renal disease
FSGS: Focal segmental glomerulosclerosis
GH: Growth hormone
IGF: Insulin-like growth factor
IGFBP: Insulin-like growth factor binding protein
IL: Interleukin
RAAS: Renin–angiotensin-aldosterone system
TNF: Tumor necrosis factor
